# Mutational networks of escape from transmitted HIV-1 infection

**DOI:** 10.1371/journal.pone.0243391

**Published:** 2020-12-07

**Authors:** Elma H. Akand, Stephen J. Maher, John M. Murray

**Affiliations:** 1 School of Mathematics and Statistics, UNSW Sydney, Kensington, NSW, Australia; 2 College of Engineering, Mathematical and Physical Sciences, University of Exeter, Exeter, United Kingdom; Consejo Superior de Investigaciones Cientificas, SPAIN

## Abstract

Human immunodeficiency virus (HIV) is subject to immune selective pressure soon after it establishes infection at the founder stage. As an individual progresses from the founder to chronic stage of infection, immune pressure forces a history of mutations that are embedded in envelope sequences. Determining this pathway of coevolving mutations can assist in understanding what is different with the founder virus and the essential pathways it takes to maintain infection. We have combined operations research and bioinformatics methods to extract key networks of mutations that differentiate founder and chronic stages for 156 subtype B and 107 subtype C envelope (gp160) sequences. The chronic networks for both subtypes revealed strikingly different hub-and-spoke topologies compared to the less structured transmission networks. This suggests that the hub nodes are impacted by the immune response and the resulting loss of fitness is compensated by mutations at the spoke positions. The major hubs in the chronic C network occur at positions 12, 137 (within the N136 glycan), and 822, and at position 306 for subtype B. While both founder networks had a more heterogeneous connected network structure, interestingly founder B subnetworks around positions 640 and 837 preferentially contained CD4 and coreceptor binding domains. Finally, we observed a differential effect of glycosylation between founder and chronic subtype B where the latter had mutational pathways significantly driven by N-glycosylation. Our study provides insights into the mutational pathways HIV takes to evade the immune response, and presents features more likely to establish founder infection, valuable for effective vaccine design.

## Introduction

After establishing HIV infection, the founder virus, which is typically monoclonal, comes under increasing immune pressure—especially from the humoral response to p24, gp41 and gp120 [1]. Regions of gp120 that form the envelope spike responsible for binding to target cells are particularly susceptible and rely on mutation to create highly variable regions to evade this response. While mutation of gp120 is necessary to avoid viral clearance, it is also constrained in the sense that the mutational pathways it follows must still allow performance of its function, namely to bind to target cells and perform the complex biomechanical steps in collaboration with gp41 necessary for insertion of the viral genome [2]. It is likely that many mutations either fail to sufficiently change its recognition by the immune response, or lead to envelope spikes that are incapable of infection. Furthermore, the virus must continue to evolve along the successful mutational pathways as immune pressure also evolves. These stepwise functional changes in the HIV envelope (Env) glycoprotein gp160 (gp120/gp41) should appear as covarying amino acid (AA) mutations embedded in Env sequences. Analysis of covarying AA differences between the earliest transmitted founder and later stage chronic HIV Env sequences may reveal these pathways. A vaccine that engenders sufficiently early blocks to these pathways may hinder evolution of the founder virus and result in HIV clearance before it is established.

In this manuscript we determine the mutational patterns that differentiate founder HIV Env sequences from their chronic counterparts. One of the difficulties in comparing sets of Env sequences arises from the high variability of regions of gp120. The five variable regions V1 to V5 encompass 14% of gp120 and include positions that are responsible for determining coreceptor usage. However, the variability of these regions makes alignment difficult to the point that analyses may largely exclude them [3]. Our previous analysis of this problem excluded positions in the alignment that were representative of insertions or deletions (indels) that consisted of gaps in more than 10% of the sequences and which were prevalent in variable regions [4]. However, these regions are important in the evolution of Env as they often lengthen through the incorporation of more N-linked glycosylation sites [5], which provide some protection against neutralizing antibodies. Current alignment methods can lead to almost random positioning of AA in variable regions that precludes a robust differentiation of Env sequences between founder and chronic groups. Accordingly, we developed a new alignment method for HIV Env (NGlyAlign) [6] that uses glycosylation sites as anchors in variable regions and produces superior results against reference alignments, as well as for the sequences investigated in this article.

Based on this more robust alignment and using a more extensive search among covarying positions, we used operations research methods to determine networks of AA positions and residues that differentiated 78 subtype B and 55 subtype C founder HIV Env sequences from approximately equal numbers of chronic sequences. These networks were formed by combining all optimal solutions that allowed separation of each group within a subtype where the fewest number of covarying AA features were used. The optimal networks were markedly different between founders and chronics regardless of HIV subtype. Network edges connect covarying AA residues and their positions (nodes), and which are contained in at least one of the optimal separating solutions. Whereas founder networks were heterogeneous with few high degree nodes, chronic networks exhibited a distinctive hub-and-spoke structure.

## Results

The inferred monoclonal transmission HIV Env (founder) sequences had been determined for 78 subtype B and 55 subtype C cases [7,8], and as previously described [9], a similar number of chronic Env sequences were obtained from the Los Alamos National Laboratory (LANL) HIV sequence database, frequency-matched to the geographical regions of the founder sequences. Including the subtype B HXB2 reference strain [10], all sequences were initially aligned using the Hidden Markov Model method HMMER [11]. Each variable region was then analysed for N-linked glycosylation sites [NXT/S] which then provided anchors via NGlyAlign [6], for the local alignment method Dialign [12,13]. The resulting multiple alignment for the subtype B sequences produced minimum entropy when compared to alignments produced by standard methods: Muscle [14], HIVAlign [15], ClustalW [16], ClustalOmega [17] and T-Coffee [18]. Better alignments will tend to match residues and so will minimise entropy scores over all positions. To allow for the lengthening of the variable regions with inclusion of indels and incorporation of glycosylation sites, the aligned Env sequences increased in length from 856 positions in HXB2 to 1144 positions. These were numbered relative to HXB2 as previously described [10].

Using this alignment, covarying pairs of AA positions were determined [4]. Covariation indicates that a residue mutation at one position of the pair possibly results in the observed AA mutation at the other position, either directly or indirectly. This produced 93,856 covarying subtype B and 86,976 subtype C pairs where the *S* measure of covariance exceeded a minimal level of 0.05. Decreasing this cut-off to 0.01 almost doubles the number of pairs but only increases the number of individual positions in the pairs by 6 (from 767 to 773) for subtype B. If there is a pair of residues (A,B) exhibited by a covarying pair with positions x-y, such that Ax-By is exhibited in some founder sequences but in no chronic sequences, then Ax-By is termed a *separating pair* for founders, and similarly when separating chronics from founders.

Operations research methods were then used to determine among the separating pairs, the fewest that could be used to separate the groups: founders from chronics, and vice versa, within each subtype. Separation of the groups is achieved when each sequence in the first group exhibits at least one of the chosen separating pairs while no sequence in the other group contains any of these. The rationale for choosing optimality based on the fewest pairs is that the features that are most biologically relevant will likely be contained in a large proportion of the sequences.

The fewest separating pairs required to separate founders (that were not in chronic) from chronics and vice-versa was 11 for subtype B and 9 for subtype C. For example, one optimal solution for founders in subtype B was I24-I415, K33-D621, S163-G164, Q183-D750, S236-Q363, K240-K340, V333-T719, L535-D620, M535-S676, R724-F837, D750-I836. This means that each founder B sequence contained at least one of these residue-pairs, while no chronic B sequence did. Although this represented an optimal solution, it was not unique; 225 other combinations of 11 covarying pairs also separated the group. This was true for each group and subtype: there were 226 optimal founder B solutions (separating them from all chronic B sequences), 125 chronic B solutions, 134 founder C solutions and 2,097 chronic C solutions. It is not surprising that there would be multiple optimal solutions. Although an optimal solution will possibly represent features which are used by the virus to evolve away from immune pressure, there can be many such pathways so that groups of patients/viruses can travel along different escape routes. Furthermore, each escape route will consist of a number of compensatory mutations that form a connected set of covarying features. The individual optimal solutions will essentially be samples from each of these routes whereas the collection of all optimal solutions may more fully describe all compensatory changes that viruses, which differ between individuals in addition to their subtype, can take in moving away from the original transmission virus. These pathways represented by the collection of all optimal solutions for each group and subtype, are described more fully below.

### Optimal coevolving networks in subtype B

#### Founder B

**Fig 1:**
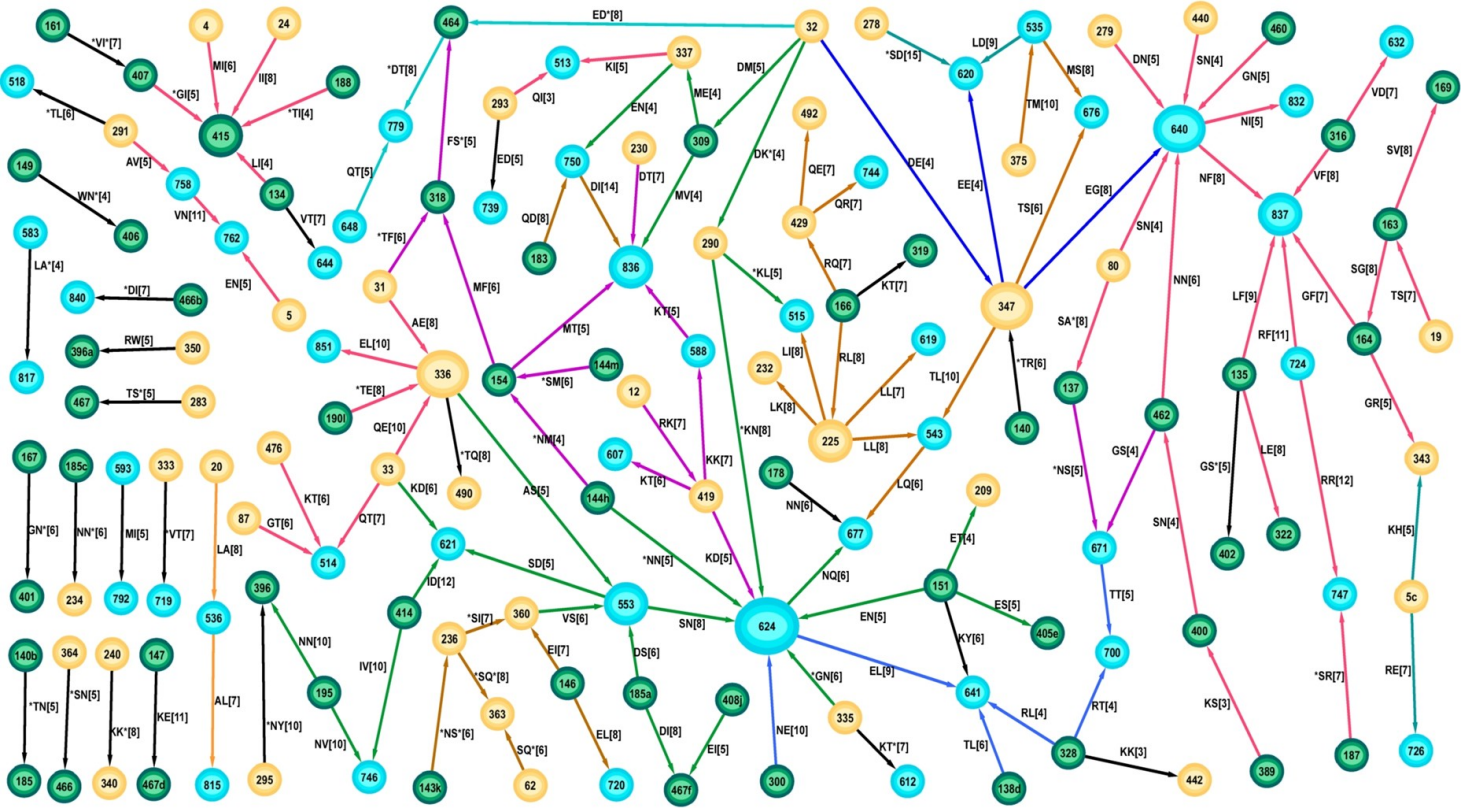
The network of all optimal features expressed by founder subtype B sequences. The feature represented by each edge is shown as a label on the edge. For example, the label RK [7] on the (directed) edge between nodes 12 and 419 denotes there were 7 sequences containing the feature R12-K419. Labels with an asterisk, eg *TI [4] for the connection between node 188 and 415, denote that the 188 node represents a position within the 3AA of a glycosylation site. Connecting edges that have at least one sequence in common so have matching AA at the node, are drawn with the same color. For example, edges incident to the node 419 have at least one common patient and the same residue Lys (K) at position 419 (R12-K419, K419-K588, K419-T607, K419-D624), and so they are all colored magenta. Nodes representing positions in the constant regions of gp120 are colored yellow, variable region nodes are green, while gp41 nodes are blue. The edges are represented as directed arrows merely to allow proper description of the node AAs.

The features within the optimal network for founder sequences describe aspects of the transmission virus that are susceptible to the eventual immune response. The immune response leads to a change so that the virus can maintain infection in the chronic state. By definition, no chronic sequence will contain any of these residue pairs. The network formed by the 226 founder B optimal solutions (Fig 1), contained 143 distinct residue-position-pairs with the most frequently occurring pair being S278-D620 with 98% of all optimal solutions containing this feature, indicating its importance in the group of 15 founders that expressed this feature (S1 Table in S1 File). This pair was incident to the glycan commencing at N276. Loss of the N276 glycan can significantly reduce clearance by broadly neutralising antibodies [19], and so it is reasonable that its presence would differentiate founder from chronic sequences. The majority (117 of 143) of the features formed a connected network with most nodes being of low degree. The highest degree node was 624 with degree 9 but this was formed by different groups of patients forming the edges incident to it; a group of 22 patients exhibited an asparagine (N) at this position, 11 patients exhibited a glutamic acid (E), while 5 sequences contained an aspartate acid (D). Although this position may be reflective of founders, its particular residue was flexible. A similar situation is observed at other high degree nodes. The large connected network was comprised of a patchwork of smaller subnetworks (as shown by the different edge colors in Fig 1) representative of sequences that exhibited consistent AA at the connecting nodes.

**Fig 2:**
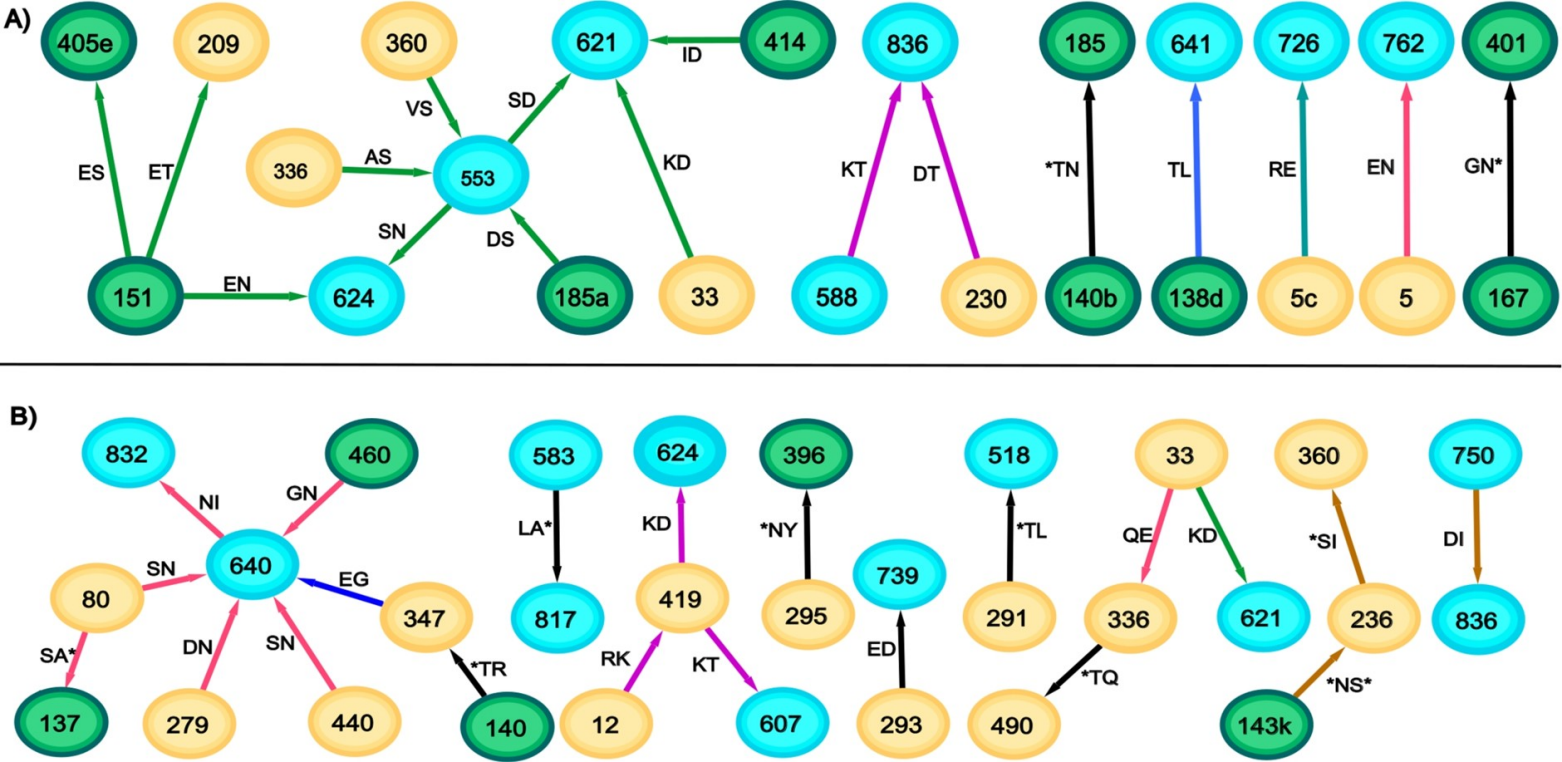
Optimal features expressed by typical founder subtype B sequences. Sequences were chosen that contained the median of 7 (A) and the maximum of 11 (B) separate components for each group.

Although the total network was largely connected, the parts expressed by the individual sequences were mostly disconnected with a median of 7 separate components (range (3,11)) (Fig 2). However the vast majority of sequences (75/78) contained features in at least one of the three subnetworks characterised either by the red subnetwork with node 640 (46/78), the green subnetwork containing 553 (58/78), or the brown subnetwork containing node 225 (in Fig 1). As shown by the coloring in the network and as noted previously [4] many (59%) of the edges were incident to positions in gp41, while 27% were incident to a glycosylation site (as described by any of its 3 positions).

#### Chronic B

**Fig 3:**
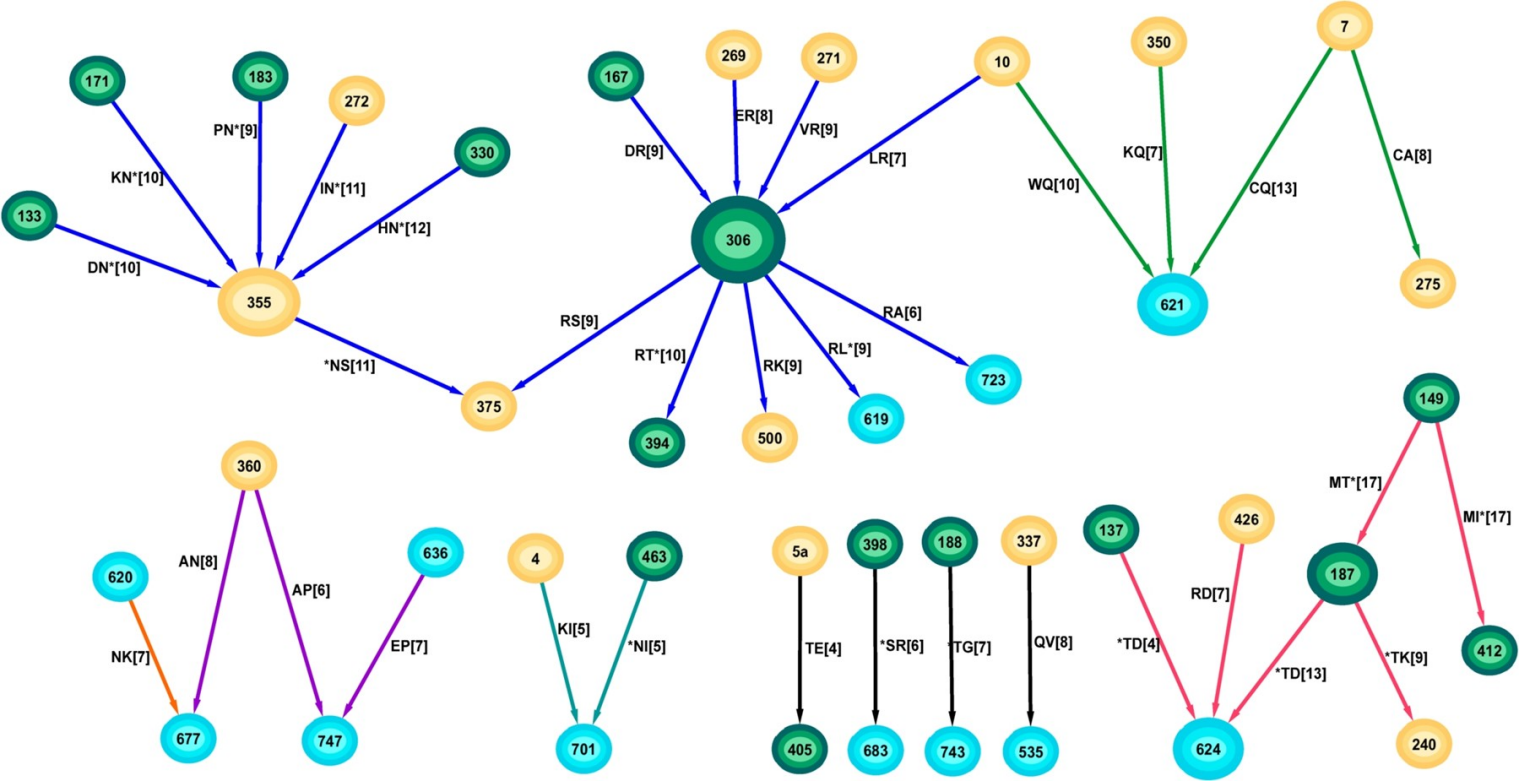
The network of all optimal chronic subtype B solutions.

Whereas features in the optimal founder networks represent characteristics of transmission away from which the virus evolves, the optimal chronic networks represent the pathways they take. The optimal founder B network consisted of 143 pairs and generally low degree nodes, however the 125 optimal chronic B solutions comprised only 35 pairs and formed a network that was highly structured, being comprised of mainly hub-and-spoke subnetworks (Fig 3).

The M149-T187 pair (part of the N-linked glycan N186) appeared in every optimal solution and covered 17 of the 78 chronic sequences (S2 Table in S1 File). It suggests that this group of chronic viruses all required this mutation pair. The glycan at position 186 significantly reduces susceptibility to the recombinant human monoclonal antibody IgG1 b12 [20], and so this pair may reflect one of the pathways to neutralisation escape. The most highly connected node was R306 with degree 9, and unlike the high degree nodes in the founder network, all edges incident to this node expressed the same arginine residue at this position. Two distinct hub-and-spoke subnetworks were identified: one centred on R306 in the V3 region and the other centred on N355 in the C3 region. Importantly, R306 (in all founder sequences the residue is serine at this position) in V3 has been identified as the single residue most indicative of a shift towards CXCR4 coreceptor tropism [21].

**Fig 4:**
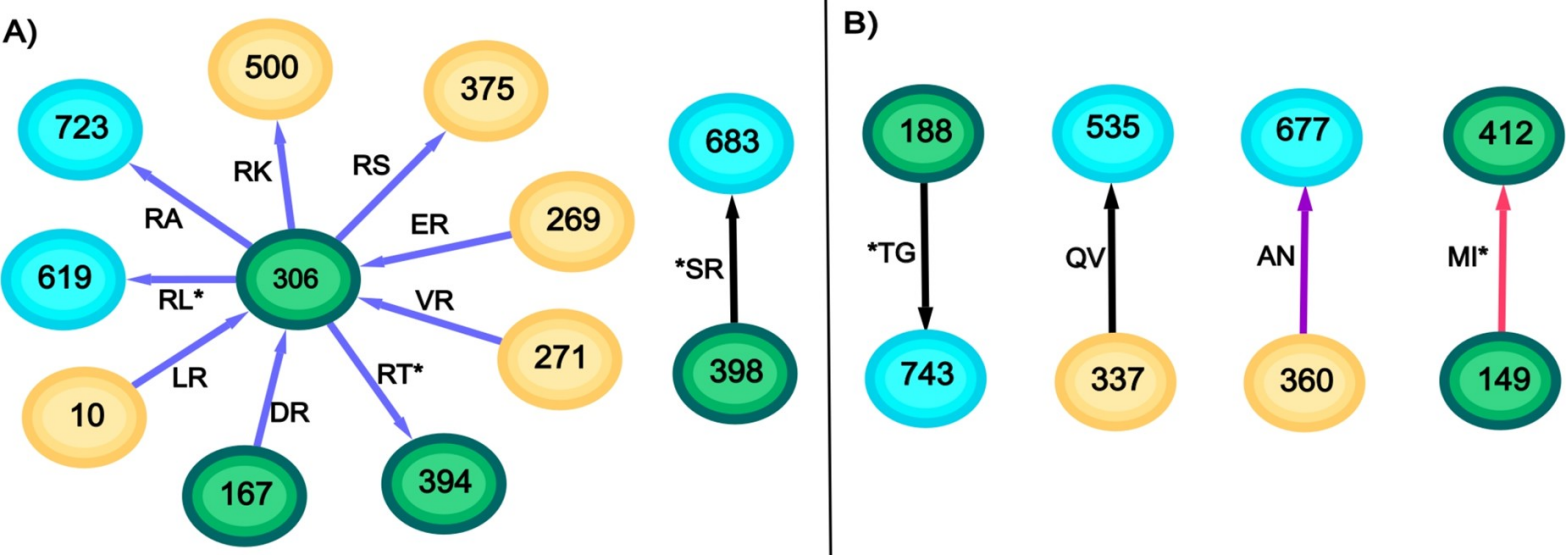
Optimal features expressed by typical chronic subtype B sequences. Sequences were chosen that contained the median (A) and maximum (B) number of separate components for each group.

The individual sequences also contained significantly fewer disconnected components than the founder B sequences (p<0.0001, Wilcoxon Ranksum Test). Whereas the individual founder sequences contained a median of 7 separate components, 91% of the chronic sequences only contained one or two separate components (median 2, range (1,4), Fig 4). More than half (43/78) of the chronic sequences contained some feature within the 6-node subnetwork containing glycans at positions 137, 187 and 412. Only 20 sequences contained features in the much larger subnetwork comprising the hubs-and-spokes around positions 306 and 355. Whereas the founder B sequences contained pairs from many of the subnetworks (only 5/78 had all separating pairs within a single subnetwork), the chronic B sequences were much more concentrated with 38/78 sequences containing separating pairs within a single subnetwork. There was a considerably higher percentage (46%) of features connecting to a glycosylation site compared to the founder B optimal network (27%, p = 0.042 Fisher Exact Test).

### Optimal coevolving networks in subtype C

#### Founder C

**Fig 5:**
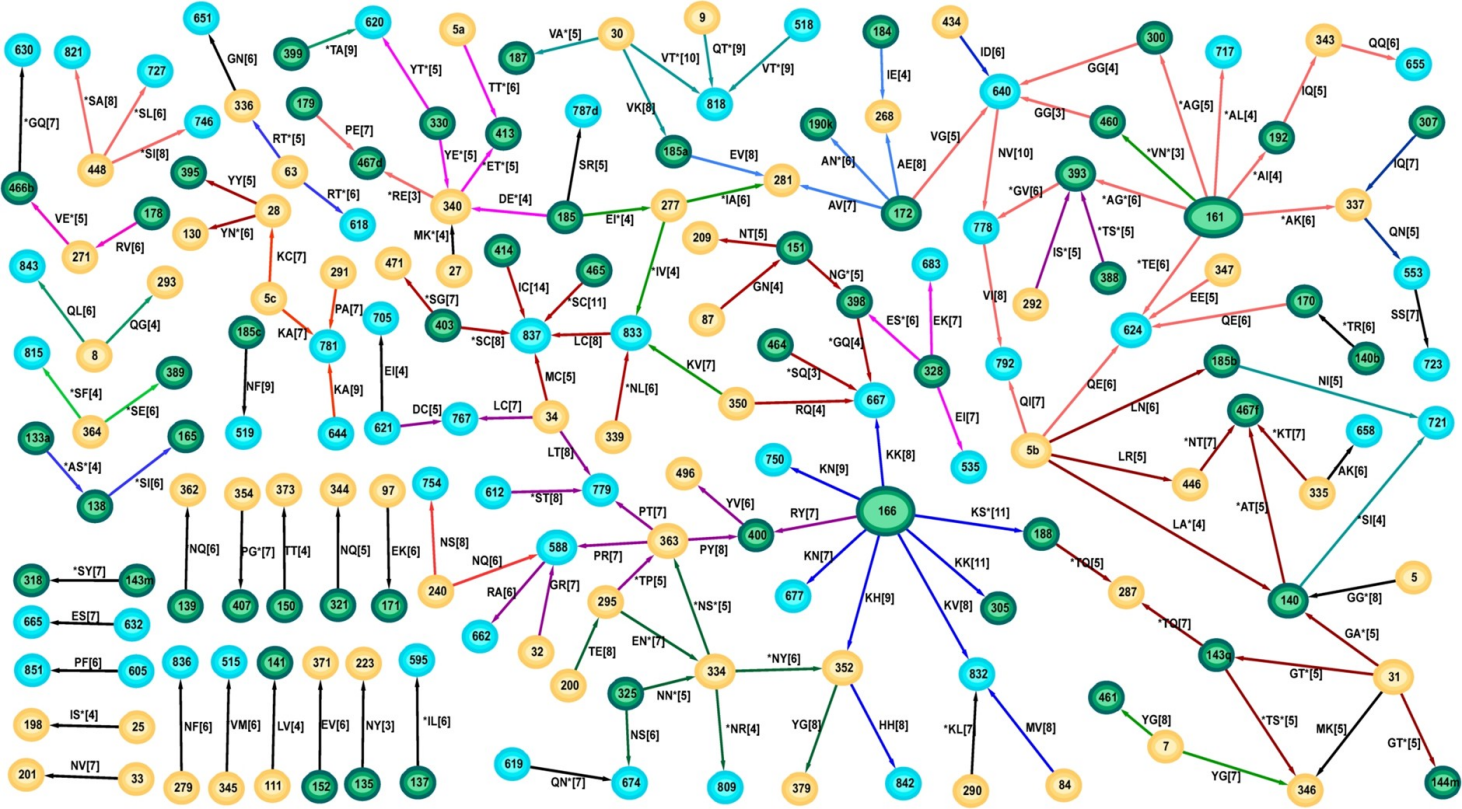
Network of optimal solutions for founder C sequences. Edges with a consistent AA at connecting nodes are shown with the same colour. These different coloured subnetworks likely represent a combination of features important in transmission for sets of viruses.

The network formed by the 134 founder C optimal solutions contained 157 distinct residue-position pairs (Fig 5). The two most frequently occurring pairs were K166-K305 and I414-C837 appearing in 55% and 53% respectively of all optimal solutions (S3 Table in S1 File). As in the founder B network, the majority (119 of 157) of features formed a connected network with most nodes being of low degree. Although this connected network contained the majority of edges, they were spread over a number of groups where the AA at the joining nodes differed as can be seen through the many colors forming the subnetworks. The highest degree node was 166 with degree 8 formed by two groups of sequences: one with a Lys(K) as in the most frequently appearing pair (K166-K305) and another group with an Arg(R), connected by the R166-Y400 pair. However, the degree 5 node at position 837 (Cys) covered most patients as 29 of the 55 sequences contained at least one feature incident to this node (24 of 78 founder B sequences were also incident to a Phe at this position). The node at position 5b covered the next most sequences (25), and combined with the 837 node, covered 41 of the 55 sequences. Individual sequences contained a median of 11 disconnected components (range (7,17), S1 Fig in S1 File). As with the founder B sequences, many of the founder C sequences shared separating pairs with individual subnetworks, where 51/55 sequences contained a separating pair in either the red subnetwork of node 161 or the green subnetwork of node 818, both of which were N-glycans. Of the pairs, 48% had an interaction with at least one gp41 site, while 48% of the pairs were glycosylated at least once. The importance of properly aligning the variable regions is underlined by 57% of these pairs having at least one site within a variable region.

#### Chronic C

There were many ways that chronic subtype C sequences could be separated from founders (2,097) but the set of different features only contained 111 covarying pairs. The combinatorial nature of the separation problem leading to the generation of so many solutions suggests that many of these features were part of the same compensatory mutational networks. There were five features that were found in at least 86% of solutions: E32-R419, H240-N448 (an N-linked glycosylation site), M4-G462, T414-A779, and I84-G500 (S4 Table in S1 File). Unlike for subtype B there was no significant difference in the number of optimal pairs incident to a glycosylation site for subtype C founders (48%), versus chronics (41%, p = 0.2 Fisher Exact Test).

**Fig 6:**
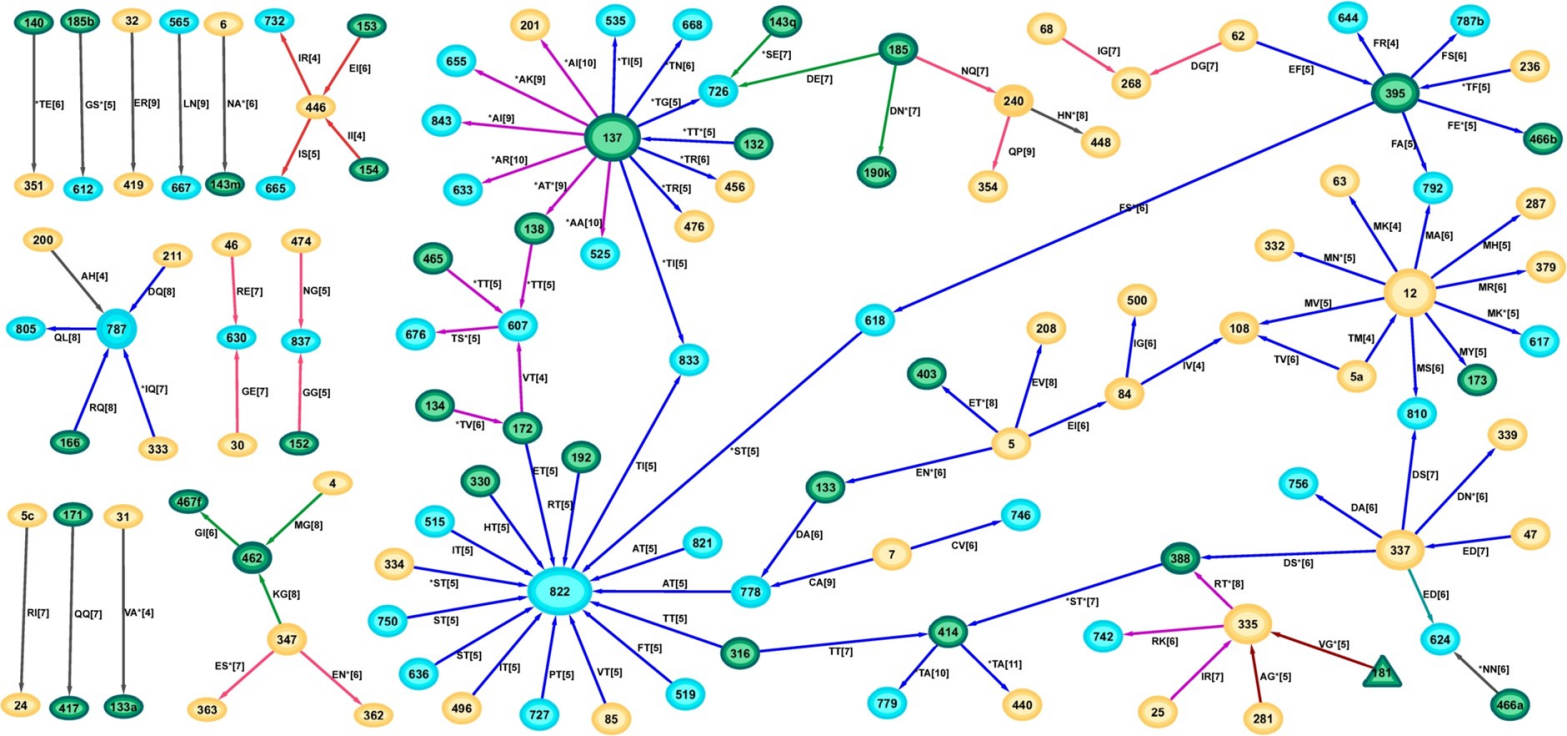
Network of optimal covarying chronic C features, coloured by consistency of joining nodes.

As for the chronic B network, the optimal chronic C features formed a highly structured network containing several hub-and-spoke components (Fig 6). The highly connected nature of some nodes in both chronic networks was reflected by their network node degrees displaying variances larger than their means (mean 1.63 and var 2.20 for B; mean 1.64 and var 3.37 for C), whereas the reverse was true for the founder networks (mean 1.55 and var 1.05 for B; mean 1.47 and var 0.80 for C, calculated for consistent AA at each node). Hence the node degrees for the founder networks could be described by a Poisson distribution but not so for the chronic node degrees which are more typical of Negative Binomial or Power Law distributions. There was one major connected subnetwork (colored blue comprising hubs at nodes 12, 137, 337, 395 and 822). Of the 52 chronic C sequences, 45 (87%) contained at least one of the features within this major subnetwork, suggesting these structures form a major pathway of viral evolution from the founder stage for this subtype. This subnetwork also contains a hub at positions 12 expressing the non-charged Met in contrast to a positively charged residue such as His that is preferentially expressed in transmission sequences [22,23]. The glycosylated hub at position 137 also formed part of the magenta subnetwork (Fig 6). The individual sequences often expressed many of the features from the larger combined network, and where they also contained some of the hub-and-spoke subnetworks (S2A Fig in S1 File). Whereas the founder C sequences tended to have many disconnected components in their individual optimal networks (median 11, range (6,17)), the chronic C sequences were highly connected as in the combined optimal network (median 5 disconnected components, range (3,9), p<0.0001 Wilcoxon Ranksum Test).

### Mapping coevolving pairs in protein 3D structures

**Fig 7:**
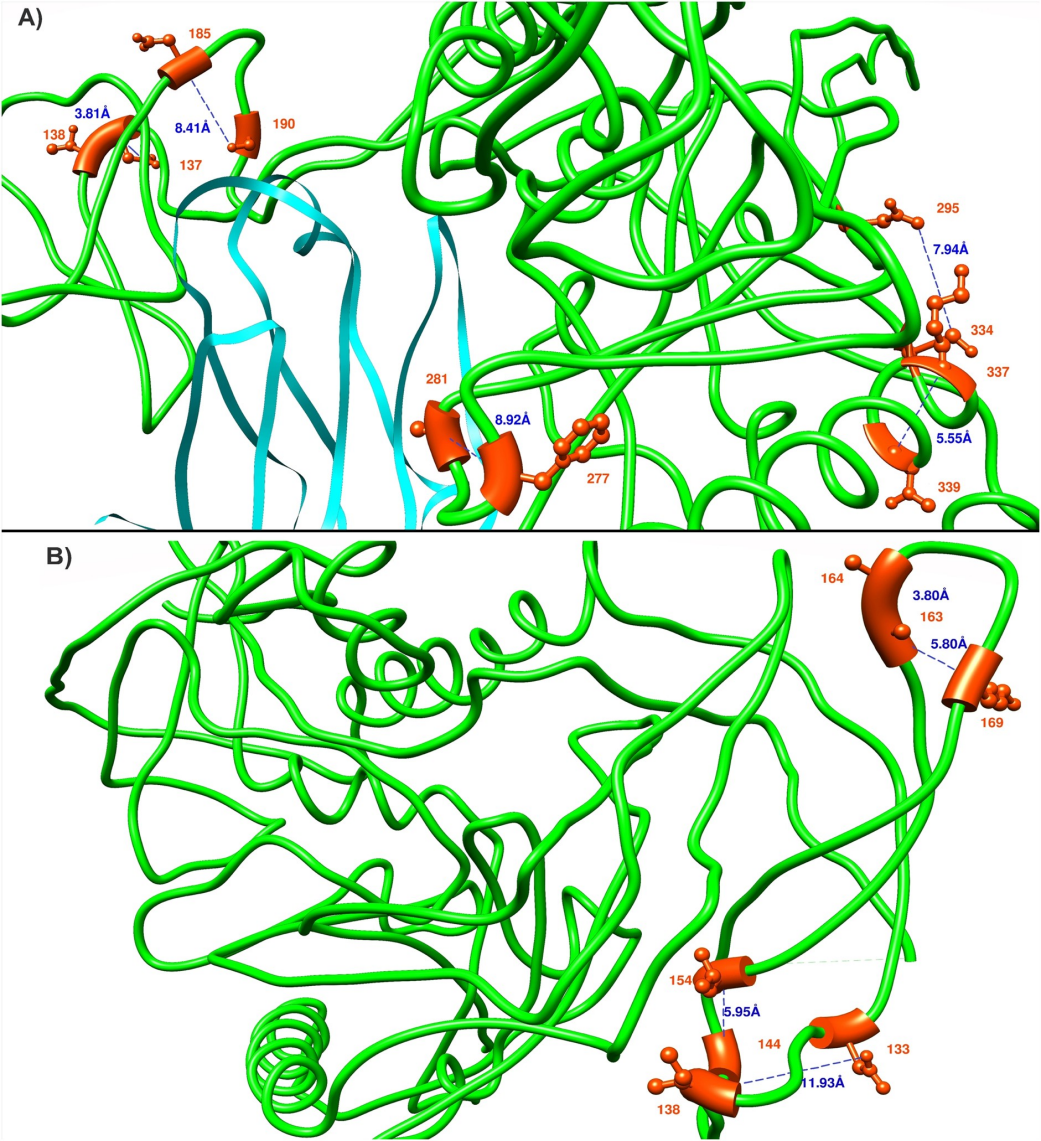
Coevolving pairs (colored as red) with minimum distance in one of the 22 structures were mapped in PDB:3J70 (A), CD4 bound, coloured cyan and PDB:3J5M (B), ligand free. All the subtype B gp120 founder pairs (S163-G164, S163-V169, S144m/h-M154) and one founder C pair (A133a-S138) were shown in PDB:3J5M. Subtype C founder pairs (I277-A281, E295-N334) and chronic pairs (A137-T138, A337-N339 and D185-N190k) were shown in PDB:3J70. The HXB2 strain was used as a reference to label the residues.

Covariance analyses can determine both functional dependence between residues as well as structural dependence [24,25]. Most of the residue pairs in the optimal networks were functionally dependent since they connected gp120 positions with those positions that could not be in direct contact, such as those in the cytoplasmic region of gp41. However, a few structurally “near” (within 8Å) residue pairs did occur as determined by comparison to 22 crystal structures. These structures included both CD4 bound (5VN3, 1G9M, 3J70, 6MEO, 3DNO, 2QAD) and unbound structures (4NCO, 5CEZ, 4TVP, 3J5M, 5UTY, 6OKP, 6E5P, 5ESV, 6MYY, 5V7J, 3U2S, 5FUU, 4R4H, 5ACO, 4ZMJ, 5VWl) in soluble and pre-soluble states. Interatomic distance was measured by computing the shortest distance among all the atoms for a residue pair. Due to the structures not describing all of Env, only 103 founder and 24 chronic coevolving pairs could be mapped to at least one of the 22 structures for subtype B, whereas for subtype C, this number was 106 for founder and 69 for chronic pairs. In total 13 pairs (5 in founder B, 4 in founder C and 4 in chronic C) were identified within 8 Å of each other (Fig 7). We note that these residues mostly appeared in adjacent or nearby positions within the sequences and they are shown (except for the pairs incident to gp41) in Fig 7, using two reference structures PDB:3J70 and PDB:3J5M. These two structures were selected as they contained the majority of the “close” coevolving pairs. The trimer undergoes significant conformational changes when changing from its ligand-free to ligand bound state so that distances between positions can vary substantially between structures.

### Sites related to CD4 and coreceptor binding

The key step for HIV-1 cell entry depends on the binding of gp120 to the host receptor CD4 and the subsequent binding to the chemokine receptors CCR5 (R5 tropic) or CXCR4 (X4 tropic). This process is mediated through the highly variable V3 loop of gp120 that recognises and binds these coreceptors. R5 tropic viruses are generally accounted for viral transmission, and X4 or dual (R5X4) tropic viruses emerge later and accelerate disease progression. Binding efficiency to these receptors depends on the spatial distribution of the participating molecules on the cell surface, dominated by the amino acid residue charge inside the binding pocket and glycosylation patterns of gp120. We determined the separating pairs incident to the CD4 binding domain (HXB2 gp160 positions 97, 102, 124, 126–128, 130, 187, 191–193, 196, 257, 279–283, 365–368, 370, 371, 425–430, 455–461, 469, 471–477), and coreceptor domains (120, 122, 193–198, 205–207, 304–309, 312, 320, 323, 324, 326, 327, 422, 423, 432, 436–440) using the structure 6MEO [26] with 5.5 Å distance cut-off. Binding irrespective of the coreceptor types, shares identical structural properties within the V3 tip residues 13–21, yet X4 viruses will be more positively charged at positions 11, 24 and 25 than their R5 tropic counterparts (the ‘11/24/25 rule’ [27]–gp160 positions 306/319/320).

**Fig 8:**
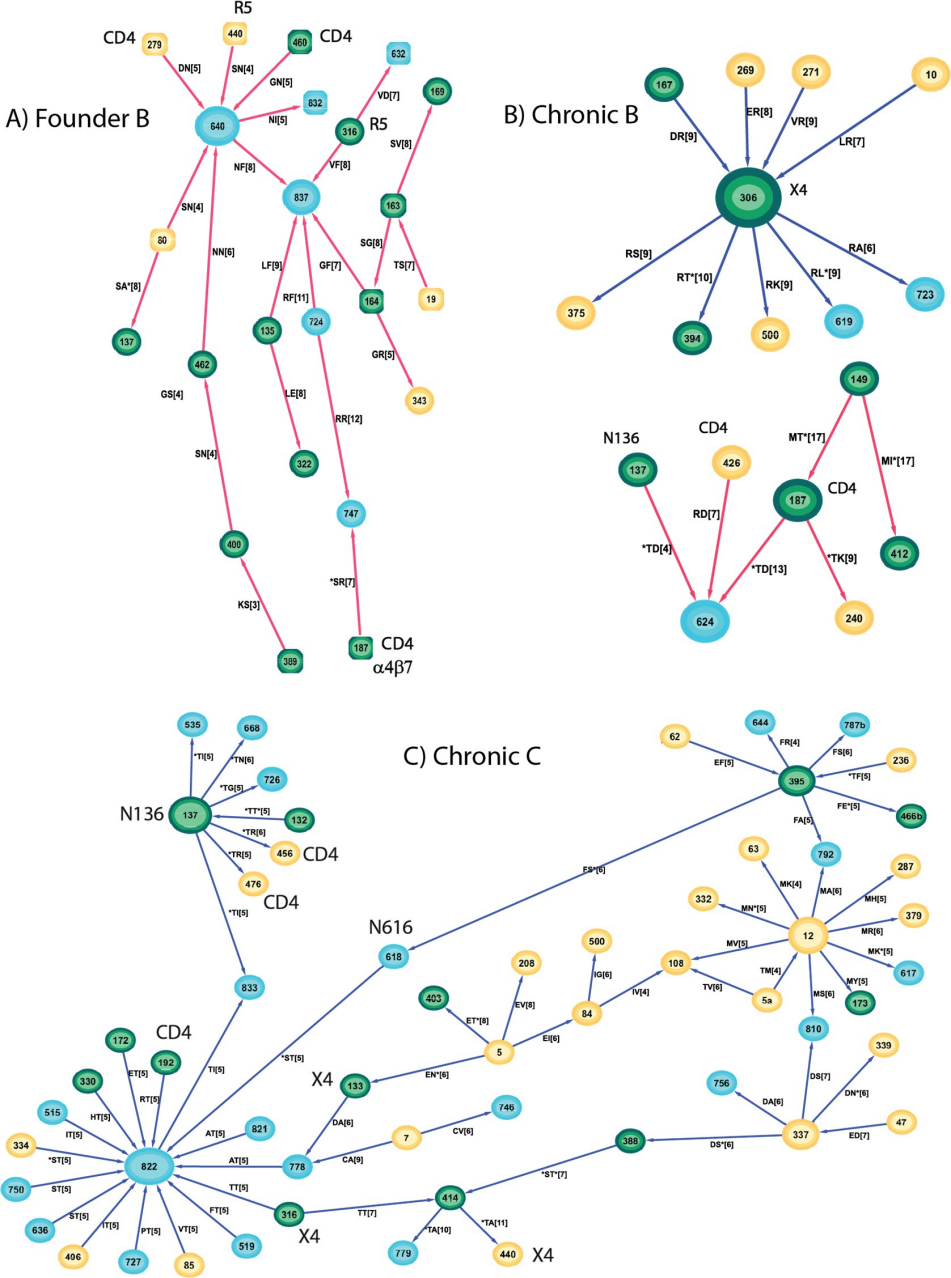
Subnetworks with preferential incorporation of CD4 and coreceptor binding domains for A) founder subtype B, B) chronic subtype B, and C) chronic subtype C. None of these domains were prevalent in any founder subtype C subnetwork.

The founder B optimal separating pairs were incident to the CD4 domain at 6 sites (*S187, D279, *T283, Q429, G460, K476) and to 5 coreceptor sites (N195, M309, V316, *F318, S440, * indicates coevolving pairs had interaction with a glycosylation site). Three of these sites (M309, V316, F318) resided at the V3 crown and all were non-polar hydrophobic residues, specific for R5 tropic virus. The founder subnetwork (red based around 640) contained the majority of these positions (3 CD4 sites and 2 coreceptor sites) suggesting the connections within that subnetwork represent aspects of binding (Fig 8A). The chronic B optimal pairs incident to the CD4 domain all reside in the one subnetwork containing *T187 and R426. Furthermore the coreceptor site R306 (positively charged) is one of the major hubs in the chronic B network, suggesting the viruses represented by this hub are more likely to be X4 or dual tropic (Fig 8B).

The founder C optimal network contained pairs incident to the CD4 domain at 10 sites (E97, *N130, *A187, *I192, N279, V/A*281, V371, G/N*460, G461, *G471) and incident to 4 coreceptor sites (K305, I307, *Y318, *S198), with no particular subnetwork being preferentially represented. Of the 5 CD4 domain sites exhibited by the optimal chronic C network, 3 (R192, R456, R476) were within the major subnetwork (blue edges and containing node 822) as were all 2 coreceptor sites (T316, A440) (Fig 8C). These sites connected through the glycosylated hub at position 136 and the hub at position 822. Unlike the chronic B coreceptor sites, there was no preference for positively charged residues at the chronic C coreceptor sites possibly reflecting less pressure in this subtype to move towards an X4-tropic phenotype, consistent with literature reports [28,29]. The remaining CD4 sites were A281 and N474.

### Phylogenetic mapping of coevolving pairs in subtype B

**Fig 9:**
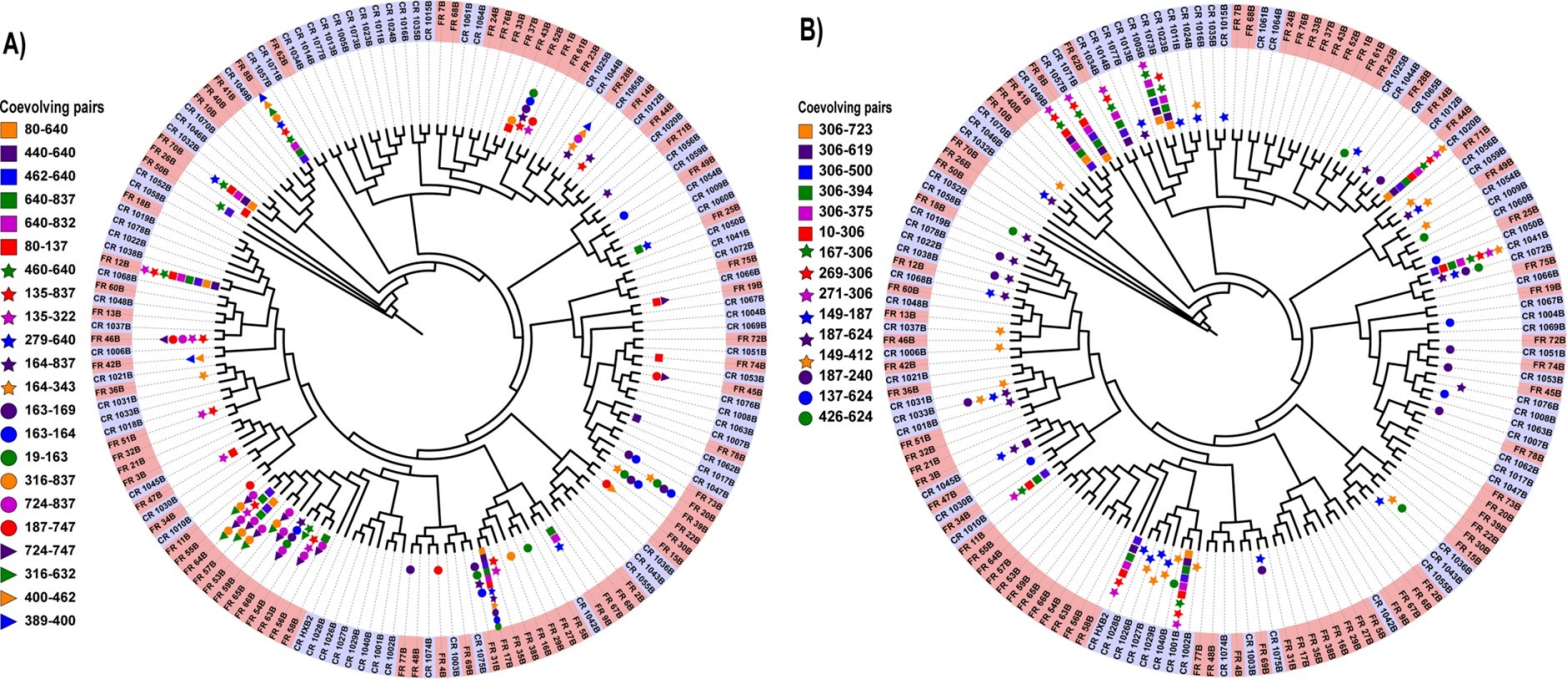
Phylogenetic trees displaying sequences that are represented within the subnetworks with preferential CD4 and CCR5 coreceptor binding: A) Founder B, and B) Chronic B. Founder sequences are shaded salmon while chronic sequences are shaded mauve.

The evolution towards these common hubs occurs for sequences that are phylogenetically distant, so that they likely represent aspects of transmission or evolution under immune pressure rather than some inherited viral similarity. The main founder B subnetwork (Fig 8A), contains 22 coevolving pairs, and the majority of edges incident to CD4 and coreceptor binding domains. The sequences that contain some of the distinctive features in this subnetwork are not necessarily close in phylogeny. The founder B hub at position 640 with CD4 and CCR5 preferential spokes represented 7 coevolving pairs and appeared within 12 sequences (8, 9, 12, 17, 25, 50, 54, 56, 57, 64, 70, 78, Fig 9A). Notably, sequences 8, 12 and 17 were phylogenetically distant, yet each contained at least 5 of the position 640 spokes and one or more CD4 binding sites (460 and/or 279) and R5 sites (316, 440). Moreover, the closest neighbours to patients 8 and 12 were chronic patients. Similarly the sequences exhibiting the features of the chronic B subnetworks that are incident to the CD4 and coreceptor binding domains are spread throughout the chronic B phylogenetic tree, including those sequences that are part of the coreceptor 306 hub (Fig 9B).

## Discussion

The most marked difference between the founder and chronic optimal networks, which represent features in one group that are not in the other, is their topology. Regardless of HIV subtype, the chronic networks contained high-degree hubs where the connecting spokes expressed a consistent residue at the hub. The most highly connected nodes in the chronic B network were R306 and N*355 (* denotes an N-glycan) of degree 9 and 6 respectively. The chronic C network contained a 16-degree hub at T822 and a 7/6 degree hub at T*/A*137 as well as a 10 degree hub at M12. Although the founder networks contained some high degree hubs, these tended to be represented by a collection of residues rather than a single one. The highest degree founder node was degree 9 for subtype B at node 624 but this consisted of 6N, 2E, and 1D, and for subtype C was 166 that expressed 7K and 1R. The presence of high degree hubs in the chronic networks suggest a concentration in evolution away from transmission virus, and that the pathways taken are relatively few. It also suggests the gp160 positions at the hubs play a dominant role in that evolution. Some of these hubs are recognisable from previous reports. The presence of a positively charged residue such as arginine at position 306 in the V3 loop for chronic B, indicates the viruses represented by this hub-and-spoke are likely to be CXCR4-tropic [29]. Similarly mutation away from a positively charged residue such as histidine at position 12 has been recognised as transitioning from a founder to a chronic virus [22,23], and has likely given rise to the M12 hub in the chronic C network. What role do the spokes play? Presumably these hub mutations carry some fitness cost that must be paid to escape the immune response, but the modifications at the spokes increase virus fitness as an offset. The evolution towards these common hubs occurs for sequences that are phylogenetically distant, so that they represent the pathways of evolution under immune pressure rather than some inherited viral similarity (Fig 9).

Founder sequences are predominantly R5-tropic [7,30] while progression through chronic infection can lead to a shift towards an X4-tropic virus [31]. We saw evidence of this differentiation in the founder versus chronic networks. Within the founder B network, 4 nodes represented positions in the CD4 binding domain and 5 nodes related to positions of coreceptor usage. The majority of these (3 CD4, 2 coreceptor), lay within the subnetwork surrounding the 640 and 827 hubs (Fig 8A), and the lack of positively charged residues at the coreceptor sites indicates CCR5 usage for the viruses that were represented within this subnetwork (46/78 sequences). On the other hand, the chronic B network exhibited a hub at position R306 indicating that this subnetwork (20/78) represents X4 or dual-tropic virus (Fig 8B). Interestingly the subnetwork covering the most sequences (43/78) was highly connected to the 2 CD4 binding sites T*187 and R426 with 4 of the 6 edges within this subnetwork incident to these CD4 sites (Fig 8B). Founder virus is less likely to contain a glycan at the α4β7 binding site (186–188) [32], whereas incorporation of the N186 glycan can confer resistance to the human monoclonal antibody IgG1 b12 specific to the CD4 binding domain [33]. Hence the networks for subtype B differentiate the majority of the viruses via coreceptor usage, but also highlight additional sites that may be important for viral fitness.

While there was no preferred subnetwork locations for the 10 CD4 and 4 coreceptor sites in the subtype C founder network, suggesting these were not dominant in characterising founder C virus, all 5 CD4 and 3 coreceptor sites for chronic C resided in the largest subnetwork covering the M12, T822 and T*137 hubs within which 45/52 sequences overlapped (Fig 8C). Although a glycosylated 137 appeared as a hub in two subnetworks, only where the residue was a threonine rather than an alanine did the subnetwork encompass the CD4 and corecoptor binding sites. As well as the concentration of CD4 and coreceptor binding domains, this subnetwork exhibited other aspects describing progression to chronic infection, M12 [22,23], and sites reflecting reduced sensitivity to broadly neutralizing antibodies (bNAbs) N136 [33]. Both chronic networks contained nodes at T*137 representing the N136 glycan; for subtype C the node was a hub for two subnetworks (T*/A*137) covering 49/52 sequences, while for subtype B the T*137 node resided in the major subnetwork containing the two CD4 binding sites at T*137 and R426. Interestingly the N*136 node did not appear in any separating pair between founders and chronics but rather this was limited to the next position in the NXT/S glycan. The other aspect of relevance to coreceptor use was the appearance of D133 in the major chronic C subnetwork. A D133A change can drastically reduce CXCR4-gp120 fusion [34], so the presence of D133 here further indicates that this subnetwork reflects a shift to an X4-tropic virus in subtype C, regardless of the neutral charge on the coreceptor residues.

Analysis of biological networks determined that as well as hubs playing a prominent role, so do bridging nodes through which much of the information flows, *C^I^* nodes [35]. The 133 node plays this role, bridging the 12 and 822 hubs and where there are 2 possible residues at that position: an asparagine that is part of an N-glycan, and an aspartic acid. So too does the 833 node connecting the hubs at 137 and 822. Position 833 lies within the lentivirus lytic peptide 1 (LLP-1, residues 828–855) within the intracytoplasmic tail of gp41. Mutations in this domain can impact viral replication and cell-cell fusion [36]. The other major bridging node in the chronic C subnetwork is 616, part of the N616 glycan. Loss of this glycan particularly impacts X4 and dual-tropic viruses [37]. Hence these bridging nodes may also contribute to the ability of these viruses to bind non-R5 coreceptors.

A comparison between founders and chronics in each subtype, confirmed that coreceptor usage was a major differentiator in our network analysis. As well as R5-usage being descriptive of founders, there were other factors in common. If hubs represent stronger aspects of each network, then a comparison of hubs between subtypes may determine similar aspects of either transmission or escape. Of all hubs degree 4 or higher, regardless of founder or chronic, only 3 hubs were common and these were confined to founders: 624 (degree 9 in B, 4 in C), 640 (8B, 5C) and 837 (5B, 5C). Nodes for 640 and 837 were directly connected within the founder B subnetwork most related to CD4 and R5 binding (Fig 8A), so both of these positions likely have strong roles in establishing infection. The 837 node in the founder C optimal network was part of the second most frequent pair C837-I414 in the optimal solutions (71/134). Of note, position 837 occurs in LLP-1 and is directly connected to another position in this domain at position 833 for the founder C network (Fig 5), and as noted above, mutations in this domain can significantly impact viral replication and cell-cell fusion [36]. Residue variability at position 640 is associated with preference for X4-tropic virus [38]. Position 624 in the pair N624-S553 was one of the pairs appearing in virus from a group of 8 subtype B seroconverters [4]. S553 in subtype B had been previously identified to contribute to trimer stability [39]. Glycosylation at N624 contributes to escape from neutralizing antibodies [37].

Although this approach has extracted features, for example, that are consistent with our understanding of coreceptor preference at different stages of disease, pointing to the validity of the approach, it has several limitations. Since operations research methods apply to a single feasible set, a fixed multiple sequence alignment (MSA) must be used. The highly variable regions within HIV Env will impact the robustness of any alignment. We therefore developed an alignment approach that has improved this process compared to other MSA [6], but this still results in uncertain alignments in some of the variable regions. Additionally, most of the edges that appear in the optimal networks are structurally distant, many of these between regions in gp120 and gp41, similar to our earlier findings [4]. Although these regions can interact to affect sensitivity to broadly neutralizing antibodies for example [40], more structurally close connections may have been expected to play a role in the Env trimer structure and therefore what differs between early and late stages of disease. That this did not occur highlights the complex linkage that mutations in the intracytoplasmic tail can have on trimer stability and its binding with CD4 and coreceptors. Notably the only hubs common to both subtypes occurred within gp41 (624, 640, 837), where these regions have previously been linked to aspects of cell-cell fusion and coreceptor usage. Our analysis reinforces the role that regions in gp41 play in maintaining the ability of the virus to infect cells against the background of an evolving immune response.

In summary, our analysis has determined aspects of HIV Env that differentiate founder and chronic virus. These results extend findings in the literature concerning evolution of the virus to usage of the CXCR4 coreceptor; not only are individual sites determined within Env responsible for this shift, but we also describe additional network structure of interactions that contribute to that change. In parallel with these coreceptor changes, immune pressure led to more highly structured hub-and-spoke networks for chronic sequences, demonstrating limited pathways that the virus can take to achieve these outcomes. The high degree features identified by these hubs may provide additional targets that limit expansion of infection away from the transmitted virus.

## Materials and methods

### Dataset and alignment

The dataset of gp160 HIV-1 subtype B and C sequences contained 263 sequences [4] of which 133 were transmission strains (78 subtype B, 55 subtype C) acquired from the study by Keele at al. [7], and Abrahams et. al. [8], with an additional 130 chronic strains (78 subtype B, 52 subtype C) randomly selected from the Los Alamos National Laboratory (LANL) HIV sequence database, as previously described [9]. By definition all of the founder sequences were antiretroviral naïve, as were 109 of the chronic sequences; the antiretroviral history of the remaining individuals was uncertain although all of these sequences were sampled at times prior to the approval of entry inhibitors (T20 (enfuvirtide) in 2003 and maraviroc in 2007). Antiretroviral usage from other classes may drive evolution of viral regions responsible for reverse transcription and protease but will not be expected to preferentially impact mutations in any aspect of Env. Where available for the chronic individuals, the mean number of CD4 cells per μL was calculated as 417 cells (standard deviation 270 cells). All the subtype B gp160 DNA sequences were translated to amino acid (AA) sequences and the set was aligned with the HIV specialized tool HMM-align [15]. This model uses HMMER [11] where a profile is built with a representative set of complete genomes of many HIV-1 subtypes and HXB2 is used as a reference.

The initial alignment was followed by identifying N-linked glycosylation sites within the variable regions and represented as a chain of conserved motifs. The segment-based alignment method DIALIGN [12,13] was used to optimize alignment as a set of conserved blocks and biologically meaningful gaps.

### Network feature generation

As previously described [4], covarying pairs of positions over the gp160 AA alignment were calculated separately for each subtype, and those pairs with a covariance calculation of *S*≥0.05 were retained. Separating pairs were determined as those covarying pairs (and their respective AA) that were expressed by at least three founder sequences but by no chronic sequences of that subtype (or vice versa). The set of separating pairs formed the feasible set for an integer programming problem that determined a subset containing the fewest number of separating pairs and for which each sequence contained at least one of these pairs [4,41]. The integer programming problem was solved with the CPLEX Interactive Optimizer 12.9.0.0. To determine all solutions for a particular problem, after each solution was determined, a constraint was introduced to exclude that solution before the next call of the optimizer. All optimal solutions had been found once the next solution could no longer achieve the minimum number of separating pairs.

Network figures were produced with Cytoscape (version 3.8.0). The structure figures were drawn with UCSF Chimera (version 1.14), while phylogenetic trees were built using the default Maximum likelihood tree with MEGA (version 10.0.4) and drawn with ITOL (version 5.5.1).

## Supporting information

S1 File (PDF)
