# Supplementary material for: Mutational networks of escape from transmitted HIV-1 infection
Source: PLoS One. 2020 Dec 7;15(12):e0243391. doi: 10.1371/journal.pone.0243391 (PMC7721145; doi:10.1371/journal.pone.0243391)
Supplement: S1 File — (PDF) [file pone.0243391.s001.pdf]

Mutational networks of escape from transmitted HIV-1 infection

Elma H. Akand[1], Stephen J. Maher[2], John M. Murray[1,*]

1 School of Mathematics and Statistics, UNSW Sydney, NSW 2052, Australia

2 College of Engineering, Mathematical and Physical Sciences, University of Exeter, Exeter EX4 4QF, United Kingdom

* Email: j.murray@unsw.edu.au

Supplementary table 1. The coevolving pairs determined on founder B that appeared in at least 10% of the 226 optimal solutions.

AA1 position	AA2 position	AA pairs	Interacting domains	Num. of observed solutions	Num. of sequences	S value
278	620	Ser-Asp	C2-gp41	222	15	0.19
553	624	Ser-Asn	gp41-gp41	198	8	0.57
147	467d	Lys-Glu	V1/V2-V5	197	11	0.18
300	624	Asn-Glu	V3-gp41	176	10	0.38
758	762	Val-Asn	gp41-gp41	172	11	0.18
724	747	Arg-Arg	gp41-gp41	134	12	0.44
464	779	Asp-Thr	V5-gp41	101	8	0.23
640	832	Asn-Ile	gp41-gp41	89	5	0.38
32	464	Glu-Asp	C1-V5	84	8	0.45
336	851	Glu-Leu	C3-gp41	71	10	0.19
335	612	Lys-Thr	C3-gp41	64	7	0.31
154	318	Met-Phe	V1/V2-V3	56	6	0.24
543	677	Leu-Gln	gp41-gp41	49	6	0.09
24	415	Ile-Ile	C1-V4	47	8	0.74
419	624	Lys-Asp	C4-gp41	36	5	0.06
750	836	Asp-Ile	gp41-gp41	32	14	0.61
144h	624	Asn-Asn	V1/V2-gp41	32	5	0.16
640	837	Asn-Phe	gp41-gp41	31	8	0.42
32	290	Asp-Lys	C1-C2	30	4	0.19
290	515	Lys-Leu	C2-gp41	30	5	1.58
419	607	Lys-Thr	C4-gp41	28	6	0.11
588	836	Lys-Thr	gp41-gp41	27	5	1.11
462	640	Asn-Asn	V5-gp41	24	6	0.45

Supplementary table 2. All 35 coevolving pairs contained in the 125 optimal chronic B solutions.

AA1 position	AA2 position	AA pairs	Interacting domains	Num. of observed solutions	Num. of sequences	S value
149	187	Met-Thr	V1/V2-V1/V2	125	17	0.58
137	624	Thr-Asp	V1/V2-gp41	119	4	0.69
350	621	Lys-Gln	C3-gp41	119	7	0.43
426	624	Arg-Asp	C4-gp41	119	7	0.41
188	743	Thr-Gly	V1/V2- gp41	119	7	0.13
337	535	Gln-Val	C3-gp41	119	8	0.72
187	240	Thr-Lys	V1/V2-C2	119	9	0.54
360	677	Ala-Asn	C3-gp41	80	8	0.18
7	621	Cys-Gln	C1-gp41	76	13	0.53
360	747	Ala-Pro	C3- gp41	45	6	0.43
7	275	Cys-Ala	C1-C2	43	8	0.32
355	375	Asn-Ser	C3-C3	26	11	0.17
330	355	His-Asn	V3-C3	26	12	0.21
171	355	Lys-Asn	V1/V2-C3	24	10	0.23
183	355	Pro-Asn	V1/V2-C3	21	9	0.13
271	306	Val-Arg	C2-V3	20	9	0.27
167	306	Asp-Arg	V1/V2-V3	20	9	0.10
306	500	Arg-Lys	V3-C5	20	9	0.62
306	394	Arg-Thr	V3-V4	20	10	0.19
272	355	Ile-Asn	C2-C3	15	11	0.12
133	355	Asp-Asn	V1/V2-C3	13	10	0.11
10	306	Leu-Arg	C1-V3	10	7	0.16
306	375	Arg-Ser	V3-C3	10	9	0.28
306	619	Arg-Leu	V3-gp41	10	9	0.07
5a	405	Thr-Glu	C1-V4	6	4	0.20
398	683	Ser-Arg	V4-gp41	6	6	0.16
636	747	Glu-Pro	gp41- gp41	6	7	0.48
620	677	Asn-Lys	gp41-gp41	6	7	0.49
269	306	Glu-Arg	C2-V3	6	8	0.41
10	621	Trp-Gln	C1-gp41	6	10	0.27
187	624	Thr-Asp	V1/V2-gp41	6	13	0.54
149	412	Met-Ile	V1/V2-V4	6	17	0.17
4	701	Lys-Ile	C1- gp41	3	5	0.07

463	701	Asn-Ile	V5-gp41	3	5	0.06
306	723	Arg-Ala	V3-gp41	3	6	0.12

Supplementary table 3. Features appearing in at least 20 of the 134 solutions for founder C sequences.

AA1 position	AA2 position	AA pairs	Interacting domains	Num. of observed solutions	Num. of sequences	S value
166	305	Lys-Lys	V1/V2-V3	74	11	0.13
414	837	Ile-Cys	V4-gp41	71	14	0.15
644	781	Lys-Ala	gp41-gp41	49	9	0.61
518	818	Val-Thr	gp41-gp41	47	9	0.16
97	171	Glu-Lys	C1-V1/V2	42	6	0.14
640	778	Asn-Val	gp41-gp41	42	10	0.43
448	821	Ser-Ala	C4-gp41	35	8	0.24
170	624	Gln-Glu	V1/V2-gp41	33	6	0.81
185c	519	Asn-Phe	V1/V2-gp41	32	9	0.10
5a	413	Thr-Thr	C1-V4	30	6	0.21
143m	318	Ser-Tyr	V1/V2-V3	29	7	0.17
335	467f	Lys-Thr	C3-V5	25	7	0.40
292	393	Ile-Ser	C2-V4	22	5	0.13
339	833	Asn-Leu	C3-gp41	20	6	0.35

Supplementary table 4. Features appearing in at least 400 of the 2097 optimal solutions for chronic C sequences.

AA1 position	AA2 position	AA pairs	Interacting domains	Num. of observed solutions	Num. of sequences	S value
32	419	Glu-Arg	C1-C4	2048	9	0.11
240	448	His-Asn	C2-C4	2010	8	0.14
4	462	Met-Gly	C1-V5	1909	8	0.64
414	779	Thr-Ala	V4- gp41	1898	10	0.06
84	500	Ile-Gly	C1-C5	1805	6	1.12
137	456	Thr-Arg	V1/V2-C4	541	6	0.16
137	668	Thr-Asn	V1/V2-gp41	537	6	0.29
333	787	Ile-Gln	C3- gp41	444	7	0.07

166	787	Arg-Gln	V1/V2- gp41	444	8	0.21
211	787	Asp-Gln	C2- gp41	444	8	0.05
787	805	Gln-Leu	gp41- gp41	444	8	0.11
137	535	Thr-Ile	V1/V2-gp41	411	5	0.20
137	833	Thr-Ile	V1/V2- gp41	411	5	0.29

Supplementary table 5. List of coevolving pairs identified within 8 Å of each other.

AA1 position	AA2 position	Min. distance	Optimal Network
A137	T138	3.7	Chronic C
A821	T822	3.8	Chronic C
D337	N339	5.2	Chronic C
D185	N190k	6.1	Chronic C
L833	C837	5.9	Founder C
I277	A281	5.9	Founder C
E295	N334	7.7	Founder C
A133a	S138	7.9	Founder C
S163	G164	3.8	Founder B
S163	V169	5.2	Founder B
V758	N762	5.9	Founder B
N144h	M154	6.0	Founder B
S144m	M154	5.9	Founder B

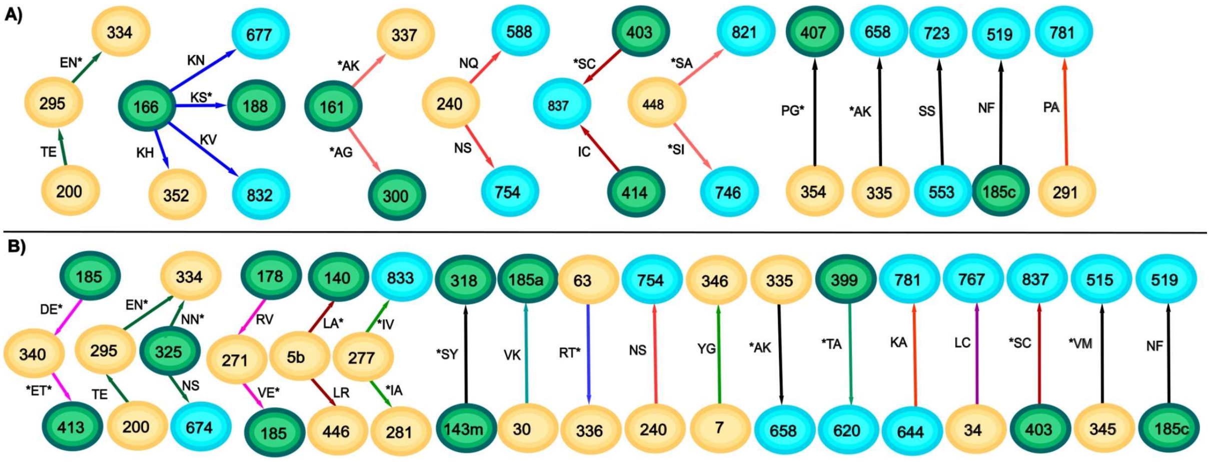

Supplementary Figure 1. Optimal features expressed by typical founder subtype C sequences. Sequences were chosen that contained the median (A) and maximum (B) number of separate components for each group.

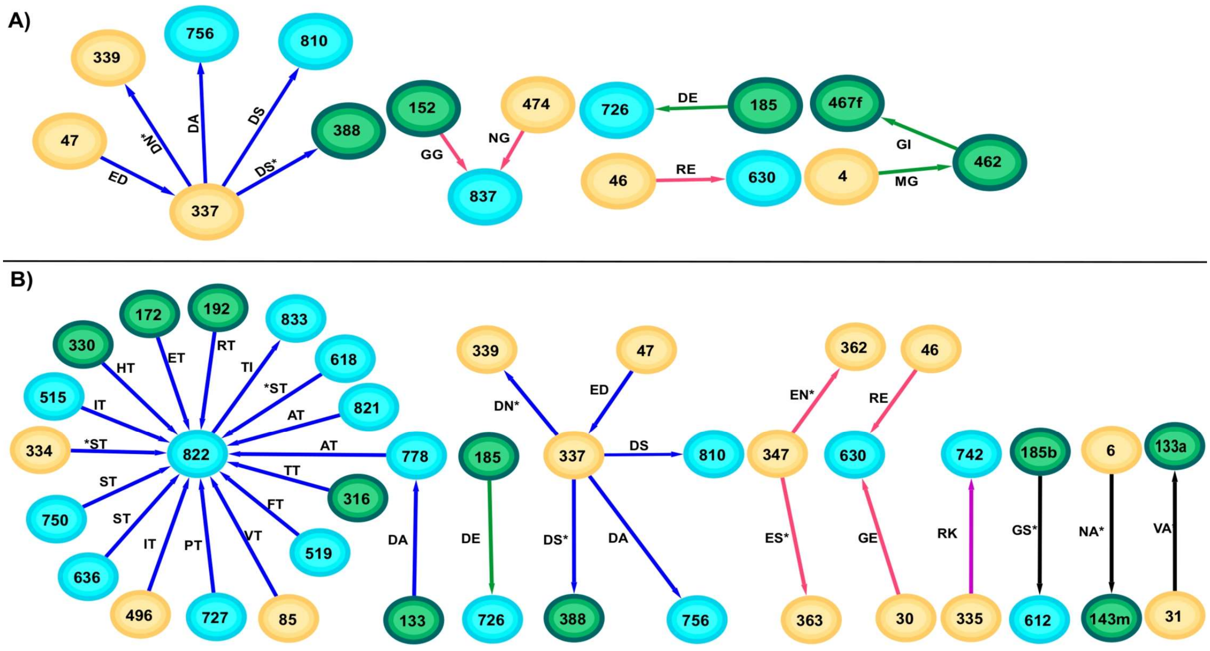

Supplementary Figure 2. Optimal features expressed by typical chronic subtype C sequences. Sequences were chosen that contained the median (A) and maximum (B) number of separate components for each group.
